# Monitoring of Hypochlorite Level in Fruits, Vegetables, and Dairy Products: A BODIPY-Based Fluorescent Probe for the Rapid and Highly Selective Detection of Hypochlorite

**DOI:** 10.1021/acsomega.3c02069

**Published:** 2023-06-09

**Authors:** Garen Suna, Eda Erdemir, Simay Gunduz, Turan Ozturk, Erman Karakuş

**Affiliations:** † Organic Chemistry Laboratory, Chemistry Group, The Scientific & Technological Research Council of Turkey, National Metrology Institute (TUBITAK UME), 41470 Gebze, Kocaeli, Turkey; ‡ Department of Chemistry, Istanbul Technical University, 34469 Maslak, Istanbul, Turkey; § Department of Chemistry, Faculty of Science, Istanbul University, 34134 Fatih, Istanbul, Turkey

## Abstract

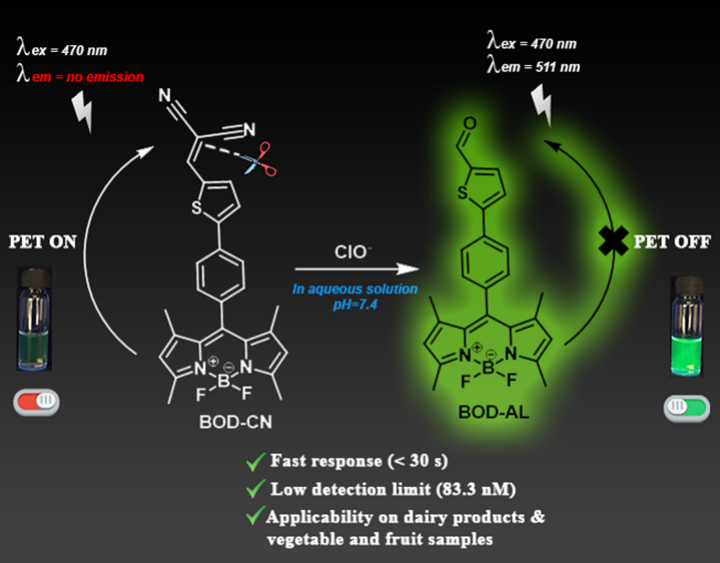

Hypochlorite/hypochlorous acid (ClO^–^/HOCl), among the diverse reactive oxygen species, plays a vital role in various biological processes. Besides, ClO^–^ is widely known as a sanitizer for fruits, vegetables, and fresh-cut produce, killing bacteria and pathogens. However, excessive level of ClO^–^ can lead to the oxidation of biomolecules such as DNA, RNA, and proteins, threatening vital organs. Therefore, reliable and effective methods are of utmost importance to monitor trace amounts of ClO^–^. In this work, a novel BODIPY-based fluorescent probe bearing thiophene and a malononitrile moiety (**BOD–CN**) was designed and constructed to efficiently detect ClO^–^, which exhibited distinct features such as excellent selectivity, sensitivity (LOD = 83.3 nM), and rapid response (<30 s). Importantly, the probe successfully detected ClO^–^ in various spiked water, milk, vegetable, and fruit samples. In all, **BOD–CN** offers a clearly promising approach to describe the quality of ClO^–^-added dairy products, water, fresh vegetables, and fruits.

## 1 Introduction

Fruits and vegetables, which are essential parts of human diet, are the sources of vitamins, minerals, carbohydrates, and fiber. They contain low fat and protein.^1^ According to the World Health Organization, insufficient consumption of fruits and vegetables can cause various diseases such as cardiovascular and some digestive system neoplasms.^2^ One of the challenges of improving fruit and vegetable consumption is extending their shelf-life.^3^ In addition to events such as transport, natural disasters, and unexpected epidemics (i.e., COVID-19), the increasing population and demand for food make fruits and vegetables having extended shelf-life more important.^4,5^

Hypochlorite (ClO^–^), which is a strong oxidizing agent, is widely used in food, vegetable, and dairy products to destroy microbial activity, delay food spoilage, and extend the shelf-life.^6,7^ Additionally, because of its critical antimicrobial properties, ClO^–^ is also used as a disinfectant for drinking water, wastewater, industrial waste treatment, and swimming pool treatment.^8−10^ On the other hand, the acceptable chlorine level ranges from 50 to 200 mg/L for fruits and vegetables, excess amount of which could damage organs and tissues resulting in several diseases such as cardiovascular, neuron degeneration, kidney, arthritis, and cancer.^11−16^ Therefore, development of a rapid and efficient method for the selective and sensitive determination of ClO^–^ is in high demand. Compared to traditional methods, fluorescent probes have many advantages, such as sensitivity, selectivity, fast response, and real-time monitoring, thus garnering tremendous attention from researchers.^17−25^ Although some fluorescent probes have been developed to detect ClO^–^, they have drawbacks, such as slow response, high detection limits, low quantum yields, turn-off response, cumbersome synthesis, and, especially, inability to be applied in actual food samples, which prompted us to develop a fluorescent probe with superior properties of selective and sensitive detection of ClO^–^ to overcome such drawbacks.^26−35^

BODIPY-based fluorescent probes have drawn extensive attention due to their important properties, such as emission at longer wavelengths, high molar absorption coefficients, high quantum yields, and versatile modification possibilities to obtain the desired properties.^36−41^

In this report, considering that the C=C double bond is readily cleaved by ClO^–^ for the use of this strategy, **BOD–CN** was synthesized from BODIPY and malononitrile via Knoevenagel condensation.^42,43^ BODIPY core was used as a signal reporter unit, and the malononitrile moiety was used as a recognition site that allowed sensitive and selective ClO^–^ detection.

This work reports a novel BODIPY-based fluorescent probe, **BOD–CN**, possessing a malononitrile moiety to detect ClO^–^ in various environments. **BOD–CN** has manifested distinct features for sensing ClO^–^, such as superior sensitivity, selectivity, and ultra-rapid response. More importantly, **BOD–CN** successfully detected the amount of ClO^–^ in complex water, disinfectant, fruit, and vegetable samples and dairy products.

## 2 Experimental Section

### 2.1 Materials and Instruments

2,4-Dimethylpyrrole (≥ 97.0, Sigma-Aldrich), 4-bromobenzaldehyde (≥ 99.0, Sigma-Aldrich), trifluoroacetic acid (≥ 99.0, Sigma-Aldrich), DDQ (≥ 98.0, Sigma-Aldrich), boron trifluoride diethyl etherate (≥ 98.0, TCI), 5-formyl-2-thienylboronic acid (≥ 95.0, Sigma-Aldrich), bis(triphenylphosphine)palladium(II) dichloride (≥ 98.0, Sigma-Aldrich), n-hexane (HPLC grade, ≥ 99.0, Sigma-Aldrich), dichloromethane (anhydrous, ≥ 99.9, Sigma-Aldrich), acetonitrile (anhydrous, ≥ 99.9, Sigma-Aldrich), ethanol (≥ 99.8, Sigma-Aldrich), chloroform (≥ 99.8, Sigma-Aldrich), and ethyl acetate (anhydrous, ≥ 99.8, Sigma-Aldrich) were used. All metal salts were used as their water-soluble sulfate, nitrate, or chloride salts, purchased from Sigma-Aldrich (≥ 95). A Varian VNMRJ 600 nuclear magnetic resonance spectrometer was used for ^1^H NMR and ^13^C NMR measurements. Mass analysis was conducted with a Thermo Q-Exactive Orbitrap device. Ultraviolet–visible (UV–vis) absorption spectra were obtained with a spectrophotometer (Shimadzu 1900i). Fluorescence emission measurements were obtained using a Varian Cary Eclipse fluorescence spectrophotometer. Quantum yield measurements were conducted with a Hamamatsu Quantaurus-QY absolute PL quantum yield spectrometer.

### 2.2 Preparation of Absorption and Emission Measurement Solutions

The stock solution of **BOD–CN** (1 mM) was prepared in a mixture of CH_3_CN and THF (1:1, v/v). The preparation of reactive oxygen species (ROS) is provided in the Supporting Information in more detail. Stock solutions of other analytes (20 mM) were prepared in triple distilled deionized water. During the measurements, other analyte solutions were added to the probe solution (2 mL) using a micropipette. The concentration of **BOD–CN** for UV–vis and emission measurements was kept at 2.5 μM in 1:1 CH_3_CN:PBS (v/v) at pH = 7.4. The samples were introduced into quartz cuvettes (1.0 cm) for fluorescence measurements. The emission spectra were recorded in the range of 485–700 nm upon excitation at 470 nm (both excitation and emission slit widths of 5 nm/5 nm). All the measurements were repeated at least three times.

### 2.3 Synthesis Section

#### 2.3.1 Synthesis of **BOD–AL**

**BOD–Br** was synthesized according to the literature procedure with slight modifications.^19^ To a mixture of **BOD–Br** (100 mg, 0.248 mmol), 5-formyl-2-thienylboronic acid (50.3 mg, 0.322 mmol), and K_2_CO_3_ (5.00 mL, 2 M) in a Schlenk tube and under nitrogen was added THF (20 mL). After the mixture was degassed, bis(triphenylphosphine)palladium(II) dichloride (17.4 mg, 0.0248 mmol) was introduced, and the mixture was heated overnight at 60 °C under a nitrogen atmosphere. Then, the mixture was filtered through celite and washed with THF. The solvent was evaporated under reduced pressure. The crude product was purified by column chromatography (hexane/DCM, 5/1) to obtain the desired product **BOD–AL** (66.7 mg, 62%) as an orange solid. ^1^H NMR (600 MHz, CDCl_3_) δ 9.92 (s, 1H), 7.82 (d, *J* = 8.0 Hz, 2H), 7.78 (d, *J* = 4.0 Hz, 1H), 7.51 (d, *J* = 3.9 Hz, 1H), 7.37 (d, *J* = 7.9 Hz, 2H), 6.01 (s, 2H), 2.55 (s, 6H), 1.44 (s, 6H). ^13^C NMR (151 MHz, CDCl_3_) δ 185.4, 158.5, 155.5, 145.6, 143.0, 140.0, 138.8, 136.3, 133.8, 131.8, 129.6, 127.3, 124.1, 17.4. HRMS (ESI): *m*/*z*: Calcd. for (C_24_H_21_BF_2_N_2_OS) [M]^+^: 434.14357; found, 434.14108.

#### 2.3.2 Synthesis of **BOD–CN**

A mixture of **BOD–AL** (50 mg, 0.11 mmol), malononitrile (22.8 mg, 0.345 mmol), and β-alanine (0.98 mg, 0.011 mmol), dissolved in a mixture of ethanol:chloroform (5 mL, 1:1 v/v), was refluxed overnight. Then, the product was precipitated in cold methanol to obtain the desired product **BOD–CN** (52 mg, 94%) as a red solid. ^1^H NMR (600 MHz, CDCl_3_) δ 7.84 (d, *J* = 8.4 Hz, 3H), 7.75 (d, *J* = 4.1 Hz, 1H), 7.53 (d, *J* = 4.1 Hz, 1H), 7.40 (d, *J* = 8.0 Hz, 2H), 5.99 (s, 2H), 2.55 (s, 6H), 1.43 (s, 6H). ^13^C NMR (151 MHz, CDCl_3_) δ 158.7, 157.4, 153.2, 145.4, 142.7, 142.6, 137.4, 135.5, 133.8, 132.0, 129.8, 127.8, 124.1, 116.6, 115.8, 17.4, 17.3. HRMS (ESI): *m*/*z*: Calcd. for (C_27_H_21_BF_2_N_4_S) [M]^+^: 482.15480; found, 482.15561.

#### 2.3.3 Synthesis of **BOD–AL** via Oxidation of **BOD–CN** with ClO^–^

To a solution of **BOD–CN** (50 mg, 0.103 mmol) in CH_3_CN/PBS (20 mL, 1:1) was added NaClO (2.06 mmol) and stirred at room temperature for 1 h. After the completion of the reaction (one spot on TLC), the solvent of the mixture was evaporated under reduced pressure. The product was purified by column chromatography using (hexane/DCM, 2/1) mixture as an eluting solvent to obtain **BOD–AL** (28 mg, 63%) as an orange solid. ^1^H NMR (600 MHz, CDCl_3_) δ 9.92 (s, 1H), 7.88–7.78 (m, 3H), 7.51 (d, *J* = 3.9 Hz, 1H), 7.45 (d, *J* = 8.2 Hz, 1H), 7.37 (d, *J* = 8.2 Hz, 1H), 6.01 (s, 2H), 2.55 (s, 6H), 1.44 (s, 6H). ^13^C NMR (151 MHz, CDCl_3_) δ 185.5, 158.5, 155.5, 145.5, 143.9, 140.0, 138.8, 136.3, 133.8, 131.7, 129.5, 127.3, 124.1, 17.4. HRMS (ESI): *m*/*z*: Calcd. for (C_24_H_21_BF_2_N_2_OS) [M + H]^+^: 435.15140; found, 435.15100.

### 2.4 Preparation of Real Samples

The recovery experiments were performed to determine ClO^–^ in pool water, spring water, wastewater, disinfectants, fruits, vegetables, and dairy products. All water samples were collected from the district of Gebze in Kocaeli Province in Turkey. To the spring and wastewater samples, a certain amount (100 μM) of various metal species, i.e., Al^3+^, Ba^2+^, Ca^2+^, Cd^2+^, Co^2+^, Cr^3+^, Cu^2+^, Fe^3+^, Hg^2+^, K^+^, Mg^2+^, Mn^2+^, Na^+^, Ni^2+^, Pb^2+^, and Zn^2+^ was introduced to obtain complex water systems. The disinfectant sample was purchased from a local market and diluted 10 times with distilled water. Similarly, fruit and vegetable samples were obtained from a local market. After obtaining the vegetable samples, i.e., strawberry, tomato, cucumber, lettuce, and spinach, they were squeezed, and their juices were centrifuged at 13,200 rpm for 10 min and filtered through a 0.45 μm membrane. Finally, the dairy products were procured from a local market, pretreated with acetic acid, and filtered with a 0.45 μm filter. The pH of all the samples was adjusted to 7.4 and stored at 4 °C. The known quantities of ClO^–^ were spiked into the juices to determine the ClO^–^ level in real samples using **BOD–CN** as a fluorescent probe (λ_em_ = 511 nm), through a linear regression equation (external calibration curve). All the experiments were repeated five times to obtain an average value of the detected ClO^–^ concentrations. Then, the recovery percentages were calculated to assess the degree of deviation of the detected value compared to the amount of added ClO^–^.

## 3 Results and Discussion

**Scheme 1:**
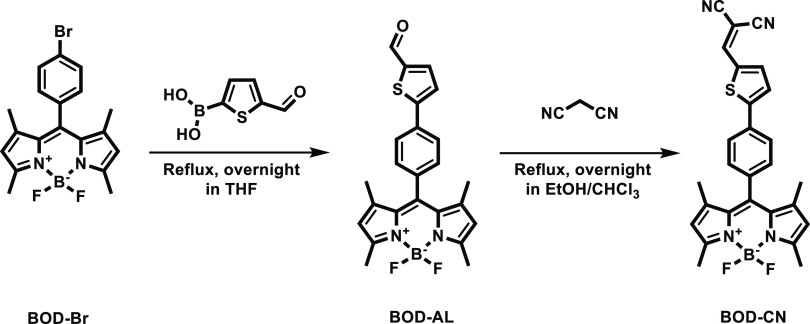
Synthetic Route of **BOD–CN**

The probe, **BOD–CN**, was prepared in a concise two-step reaction (Scheme 1). Suzuki coupling of the readily available **BOD–Br**(36) with the commercially available 5-formyl-2-thienylboronic acid produced the aldehyde **BOD–AL**, Knoevenagel condensation of which with malononitrile gave the desired probe **BOD–CN**. Its structure was confirmed by ^1^H NMR, ^13^C NMR, and HRMS techniques (see Supporting Information).

Initially, the ideal solvent system was determined by examining various solvent systems, i.e., DMSO–H_2_O, EtOH–H_2_O, THF–H_2_O, and CH_3_CN–H_2_O, among which CH_3_CN–H_2_O was found to be the most effective system (Figure S1). When the amount of CH_3_CN in the mixture was less than 50%, fluorescence intensity was dramatically diminished, and the mixture of CH_3_CN–H_2_O (1:1, v/v) was found to be the best solvent system (Figure S2). The probe showed remarkable stability over a wide pH range (pH = 2–10), and the sensing occurred in a pH range of 6–10. Then, the pH of the reaction medium was set to 7.4, and a phosphate buffer solution (PBS, 10 mM) was used under physiological conditions (Figure S3).

**Figure 1:**
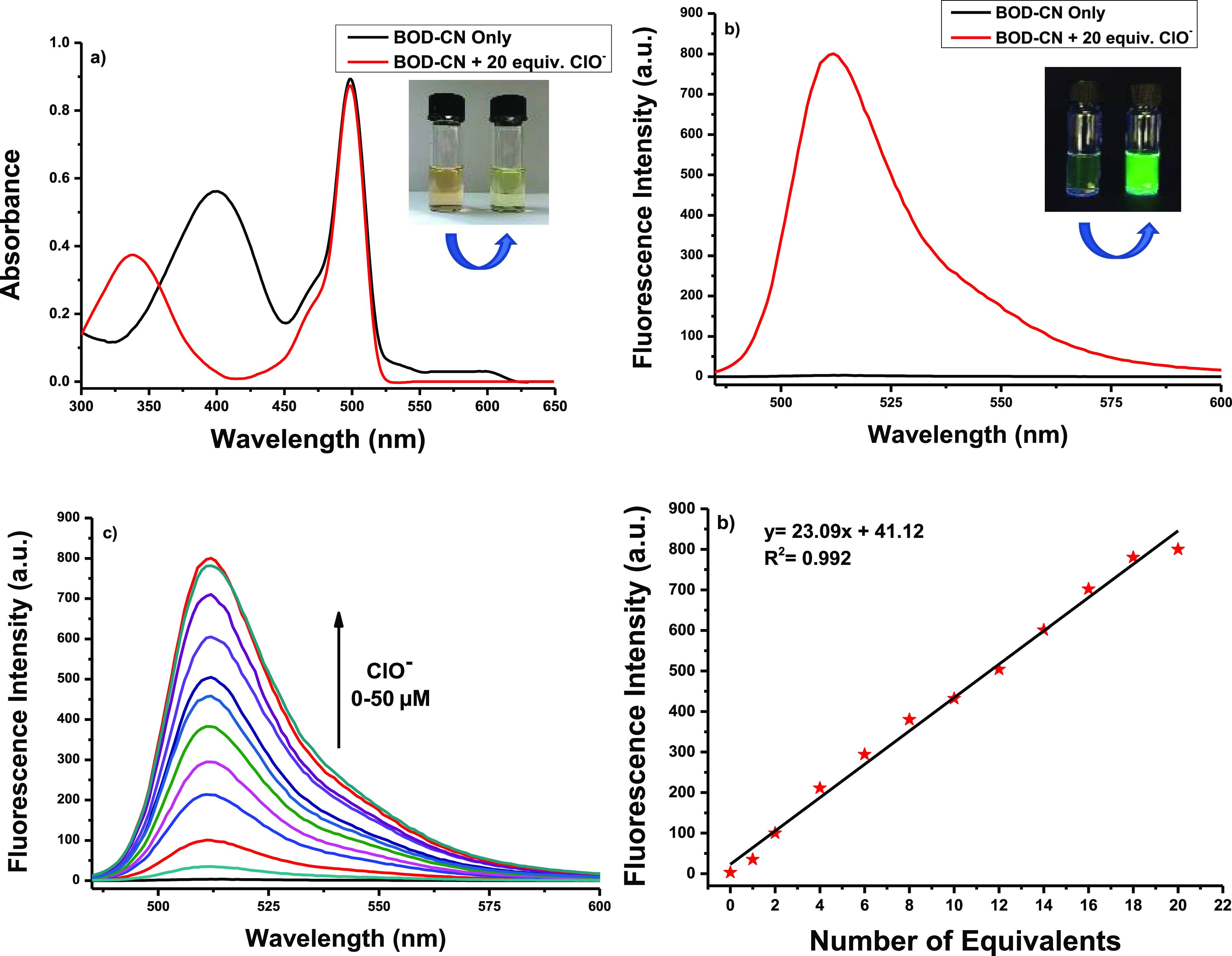
(a) Absorption and (b) emission spectra of **BOD–CN** (2.5 μM) and ClO^–^ (20 equiv, 50 μM) in 1:1 CH_3_CN/PBS at pH = 7.4. (c) Fluorescence titration spectra of **BOD–CN** (2.5 μM) in 1:1 CH_3_CN/PBS at pH = 7.4 in the presence of ClO^–^ (0–20 equiv, 0–50 μM). (d) Fluorescence intensity changes depending on the mole equivalents of ClO^–^ = 0–20 equiv.

The optical responses of **BOD–CN** in the absence and presence of ClO^–^ were measured. The absorption peak at 400 nm could be attributed to the push–pull effect of BODIPY through the malononitrile moiety. However, the addition of ClO^–^ to **BOD–CN** led to the formation of a new product (**BOD–AL**) which caused a blue shift in the absorption maxima from 400 to 332 nm with a slight decrease in absorbance intensity. The color of the solution became light yellow in a few seconds, easily observable with the naked eye (Figure 1a).

As we expected, the free **BOD–CN** was nonfluorescent (φ_F_ = 0.02) due to photoinduced electron transfer (PET) from the donor part of the molecule (BODIPY) to the acceptor part of the molecule (malononitrile). The PET process was blocked upon the addition of ClO^–^ to the **BOD–CN** solution, and a new emission (φ_F_ = 0.36) peak became centered at 511 nm in the fluorescence spectrum (Figure 1b). The fluorescence intensity achieved a plateau with the addition of 20 equiv ClO^–^ for a 200-fold enhancement (Figure 1c,d). Although the fluorescence response was rapid (<30 s), a complete saturation was observed after 5 min (Figure S4). The detection limit of **BOD–CN** for ClO^–^ was calculated to be 83.3 nM, based on the linear regression analysis (Figure S5 and Table S1).

**Figure 2:**
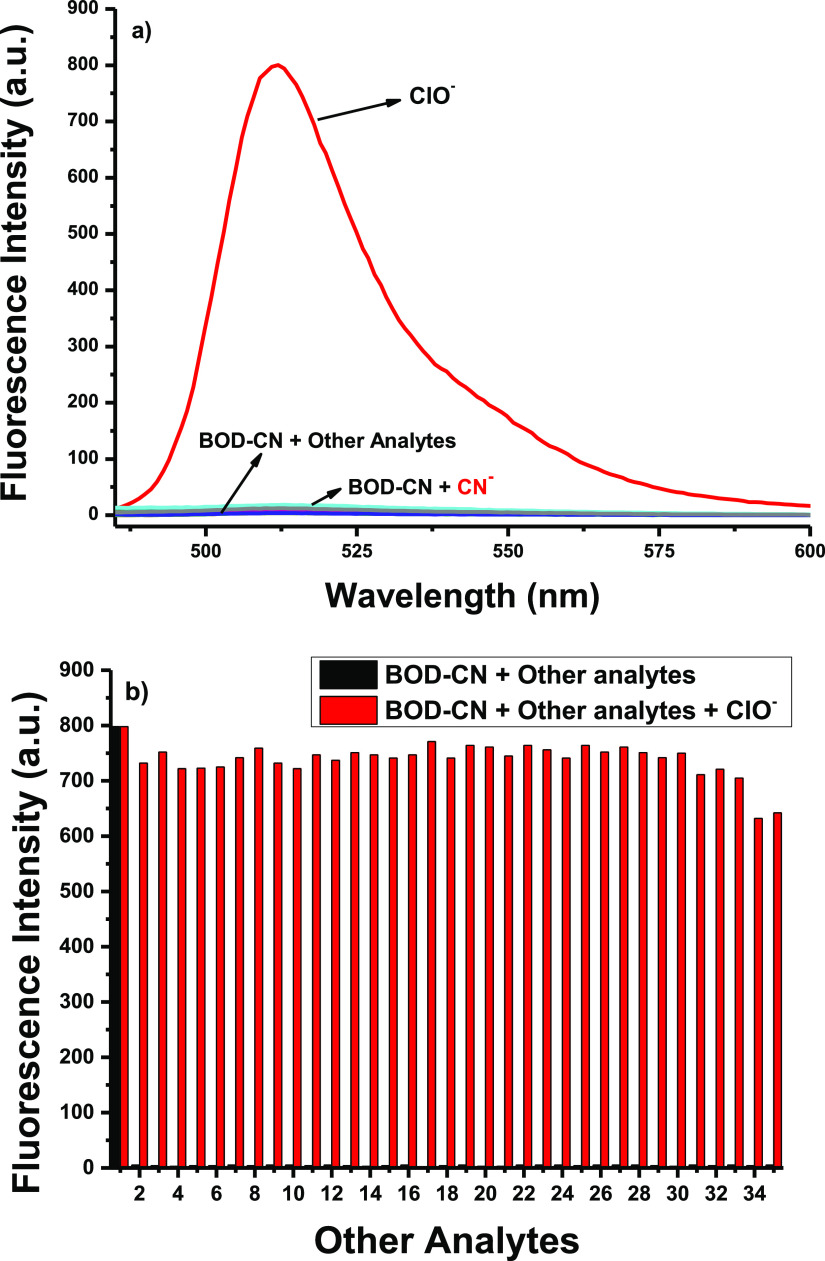
(a) Fluorescence intensities of **BOD–CN** (2.5 μM) in 1:1 CH_3_CN/PBS at pH = 7.4 at λ_max_: 511 nm in the presence of ClO^–^ (20 equiv) and other analytes (100 equiv) or ROS (20 equiv) separately. (b) Fluorescence intensities of **BOD–CN** (2.5 μM) in 1:1 CH_3_CN/PBS at pH = 7.4 in the presence of ClO^–^ (20 equiv) and other analytes (100 equiv) or ROS (20 equiv) together: 1, ClO^–^; 2, F^–^; 3, Cl^–^; 4, Br^–^; 5, I^–^; 6, NO_3_^–^; 7, N_3_^–^; 8, HCO_3_^–^; 9, AcO^–^; 10, ClO_4_^–^; 11, PO_4_^2–^; 12, SO_3_^2–^; 13, SO_4_^2–^; 14, PO_4_^3–^; 15, Na^+^; 16, K^+^; 17, Ca^2+^;18, Mg^2+^ ;19, Hg^2+^; 20, Pb^2+^; 21, Cys; 22, Hcy; 23, GSH; 24, H_2_S; 25, l-valine; 26, l-alanine; 27, l-arginine; 28, l-phenylalanine; 29, l-glycine; 30, l-lysine; 31, HO^·^; 32, NO^·^; 33, TBHP; 34, ROO^·^; and 35, O_2_^·^^–^.

As the selectivity is an indispensable parameter for an efficient fluorescent probe, **BOD–CN** was tested for ClO^–^ in the presence of various analytes. Thus, the addition of 100 equiv of other analytes, i.e., F^–^, Cl^–^, Br^–^, I^–^, NO_3_^–^, N_3_^–^, HCO_3_^–^, AcO^–^, ClO_4_^–^, CN^–^, PO_4_^2–^, SO_3_^2–^, SO_4_^2–^, PO_4_^3–^, Na^+^, K^+^, Ca^2+^, Mg^2+^, Hg^2+^, Pb^2+^, Cys, Hcy, GSH, H_2_S, l-valine, l-alanine, l-arginine, l-phenylalanine, l-glycine, l-lysine, and 20 equiv of ROS such as hydroxyl radical (HO^·^), hydrogen peroxide (H_2_O_2_), tert-butyl hydroperoxide (TBHP), superoxide radical (O_2_^^·^–^), peroxyl radical (ROO^·^), and nitric oxide (NO^·^) did not cause any change in the fluorescence spectrum (Figure 2a). The malononitrile unit is commonly used with various fluorophores for the detection of CN^–^ ions.^44−49^ Although it is a highly popular and known strategy for that purpose, surprisingly, in our sensing system, CN^–^ ions did not induce any alteration in the fluorescence intensity. This result showed that our sensing strategy has outstanding selectivity over other reported studies in the literature (Figure 2a). Then, to prove that the probe is unaffected by the presence of other analytes, fluorescence measurements were recorded by adding 20 equiv of ClO^–^ in the presence of 100 equiv of other analytes. The evaluation revealed that **BOD–CN** could smoothly detect ClO^–^ even in the presence of other competing analytes in high concentrations (Figure 2b).

**Scheme 2:**
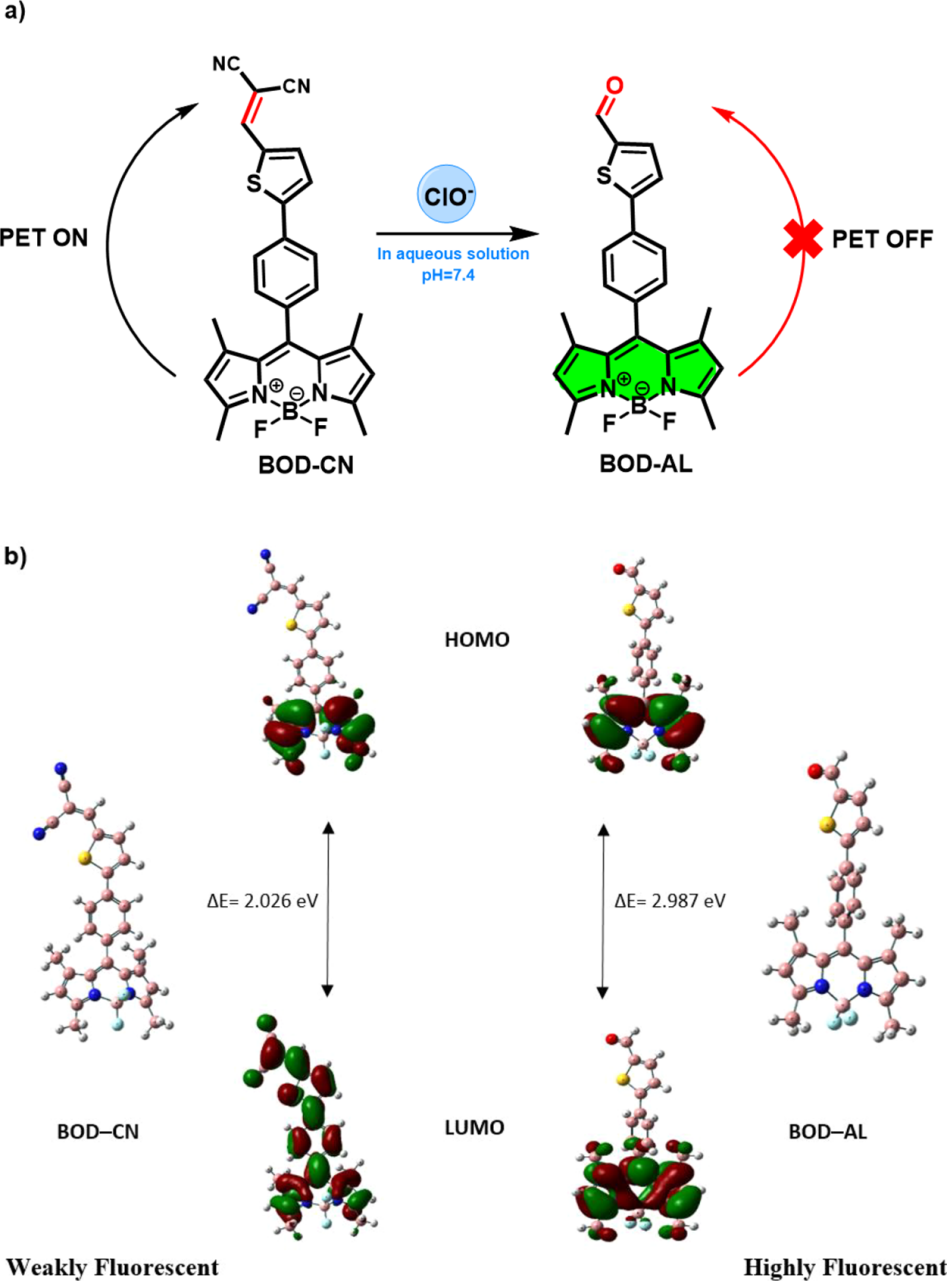
(a) Proposed Mechanism for the Detection of ClO^–^; (b) Optimized Structures and the Molecular Orbitals of **BOD–CN** and **BOD–AL**

The sensing mechanism of **BOD–CN** was investigated through computational studies before and after the addition of ClO^–^. Density functional theory (DFT) calculations at B3LYP/6-31G(d,p) level were used for the optimization of the ground states (Scheme 2).

While the electrons of the highest occupied molecular orbital (HOMO) of **BOD–CN** were localized on the BODIPY unit, they were delocalized significantly over the thiophene and dicyano-vinyl groups at LUMO, suggesting a PET from BODIPY to the dicyano-vinyl group. On the other hand, addition of ClO^–^ resulted in the localization of the electrons on the BODIPY unit at both the HOMO and LUMO of **BOD-AL**. An increase in the fluorescence intensity after the oxidation of the dicyano-vinyl moiety also correlated with the localization of the electrons on BODIPY and the inhibition of the PET process. This explains the sensing mechanism of formation of aldehyde upon oxidation of the malonitrile unit with ClO^–^, which was also proved by HRMS and NMR analyses of the probe solution (BOD–CN + ClO^–^). Detection of the molecular ion peak of the aldehyde **BOD–AL**, i.e., *m/z* 434.14203, indicated the oxidation of nonfluorescent **BOD–CN** to produce fluorescent **BOD–AL** (Scheme 2a, see Supporting Information).

Encouraged by the outstanding analytical performance and optical features of the probe **BOD–CN** toward ClO^–^ detection, its applications in spring water, wastewater, pool water, dairy products, and disinfectants were examined. Since the improper release of industrial wastes containing ClO^–^ can pollute water resources, and the increase in the use of ClO^–^ in foods and dairy products reduces the microbial activity, it is highly important to develop appropriate methods for monitoring the presence of ClO^–^ in those samples. Therefore, after adding the known quantities of ClO^–^ to the water samples, dairy products, and disinfectants, an external calibration curve was created for each sample containing 2.5 μM **BOD–CN** separately to observe the matrix effect (Figures S6 and S8). Recovery values were calculated to be in the range of 98.1–100.5%. The recovery values and relative standard deviations (RSDs) between 0.40 and 3.58% demonstrated that the probe **BOD–CN** accurately detects the amount of ClO^–^ in complex water samples, dairy products, and disinfectants (Table 1).

**Table 1:** Results for the Detection of ClO^–^ in Different Water Samples and Disinfectants

samples	ClO^–^ spiked (μM)	ClO^–^ found (μM)	recovery (%)	RSD (%) (*n* = 5)
spring water	12.50	12.41 ± 0.05	99.3	0.40
wastewater	12.50	12.34 ± 0.15	98.7	1.22
pool water	12.50	12.26 ± 0.18	98.1	1.47
disinfectant	12.50	12.21 ± 0.16	97.7	1.31
milk	25.00	24.86 ± 0.45	99.4	1.81
yogurt	25.00	25.13 ± 0.90	100.5	3.58

**Figure 3:**
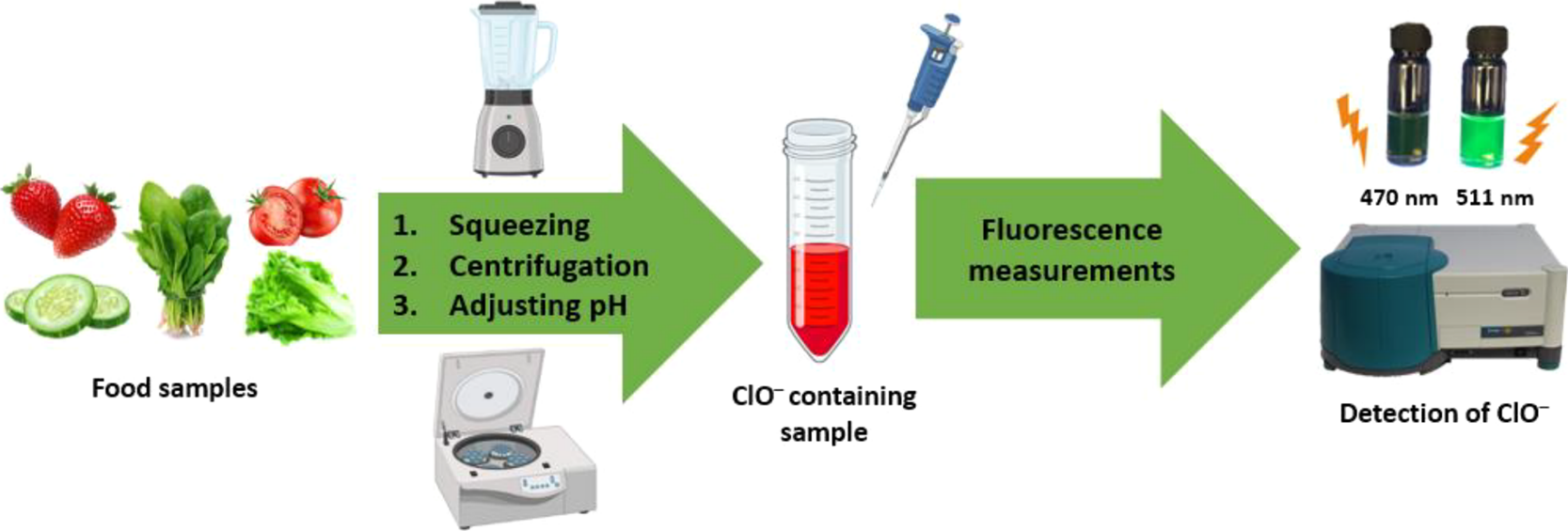
Sensing pathway of ClO^–^ contents with the **BOD–CN** probe in fresh food samples.

The ability of **BOD–CN** to determine the amount of ClO^–^ in different fruit and vegetable samples was investigated. Washing fruits and vegetables with ClO^–^ solution prolongs their shelf-life by slowing down microbial activities on their surface. However, any possible ClO^–^ residue can endanger human health. For this reason, monitoring the amount of ClO^–^ in fruits and vegetables is essential. Thus, known quantities of ClO^–^ were added into 10 μM **BOD–CN** samples to generate an external calibration curve for each sample (Figures 3 and S7) to observe the matrix effect. Recovery values were calculated to be between nearly 98.0 and 102.0%, indicating that **BOD–CN** can feasibly determine the amount of ClO^–^ in various fruit and vegetable samples (Table 2).

**Table 2:** Results for the Detection of ClO^–^ in Fruit and Vegetable Samples

samples	ClO^–^ spiked (μM)	ClO^–^ found (μM)	recovery (%)	RSD (%) (*n* = 5)
strawberry	25.00	25.09 ± 0.75	100.4	2.99
tomato	25.00	25.35 ± 0.73	101.4	2.88
cucumber	25.00	25.52 ± 0.32	102.1	1.25
lettuce	25.00	24.79 ± 0.76	99.2	3.07
spinach	25.00	24.63 ± 0.67	98.5	2.72

## 4 Conclusions

A novel BODIPY-based fluorescent probe **BOD–CN** was designed and synthesized for rapid, sensitive, and selective ClO^–^ detection. The probe demonstrated outstanding features of low detection limit (83.3 nM), superior selectivity, and rapid response (<30 s). The sensing mechanism of **BOD–CN** was explained to rely on the aldehyde formation by the cleavage of the C=C bond through oxidation with ClO^–^. The application of the probe to ClO^–^-spiked water, disinfectants, fruits, vegetables, and dairy products gave satisfactory results, demonstrating great sensitivity toward ClO^–^ in actual food samples. In summary, the sensing mechanism of **BOD–CN** offers a fast, reliable, and convenient method for ClO^–^ detection in various samples in daily life.

## Supporting Information Available

Preparation of reactive oxygen species (ROS); optimization studies; calibration curves of the sample application; comparison with other reported ClO^–^ fluorescent probes; and ^1^H NMR, ^13^C NMR, and HRMS measurements (PDF)
