# Supplementary material for: Monitoring of Hypochlorite Level in Fruits, Vegetables, and Dairy Products: A BODIPY-Based Fluorescent Probe for the Rapid and Highly Selective Detection of Hypochlorite
Source: ACS Omega. 2023 Jun 9;8(25):22984–91. doi: 10.1021/acsomega.3c02069 (PMC10308583; doi:10.1021/acsomega.3c02069)
Supplement: Supplementary file 1 — ao3c02069_si_001.pdf [file ao3c02069_si_001.pdf]

Monitoring of Hypochlorite Level in Fruits, Vegetables and Dairy Products: A BODIPY-based Fluorescent Probe for the Rapid and Highly Selective Detection of Hypochlorite

Garen Suna [a, b, 1], Eda Erdemir [a, c, 1], Simay Gunduz [a], Turan Ozturk [a, b], Erman Karakuş *[a]

[a]Organic Chemistry Laboratory, Chemistry Group, The Scientific & Technological Research Council of Turkey, National Metrology Institute, (TUBITAK UME), 41470 Gebze, Kocaeli, Turkey erman.karakus@tubitak.gov.tr
[b]Department of Chemistry, Istanbul Technical University, 34469, Maslak, Istanbul, Turkey
[c]Department of Chemistry, Faculty of Science, Istanbul University, 34134, Istanbul, Turkey

[1] Both authors contributed equally to this work

CONTENTS **PAGE**

1. Preparation of Reactive Oxygen Species (ROS)……………………………………………….2

2. Fluorescence Spectrum of **BOD–CN** with Different Solvent Systems……….………………..…3

3. Effect of Phosphate Buffer Solution (PBS) Content in Sensing Mechanism ……….….……..…3

4. Effect of pH ………………………………………………..…...…………………………...4

5. Time-Dependant Fluorescence Change of **BOD–CN** with ClO^{-}. ....….……………………..…...4

6. LOD Determination of ClO^{-}………………………..……………………………………..…5

7. Calibration Curves of Real Water Samples and Disinfectant….………………………………....5

8. Calibration Curves of Fruit and Vegetable Samples……………..…...…………………………..…7

9. Calibration Curves of Dairy Products………………..………….…...…………….…………..…9

10. Comparison table with other reported ClO^{-} fluorescent probes……………….………………10

^{1}H and ^{13}C NMR of **BOD–AL** ..…………....……………………………………………………13

^{1}H and ^{13}C NMR of **BOD–CN** ………….....…………………………………………………..14

^{1}H and ^{13}C NMR of **BOD–AL** after oxidation with ClO^{-}……………………………………………..15

HRMS spectrum of **BOD–AL** ……………..……………………………………………………16

HRMS spectrum of **BOD–CN** ……………..……………………………………………………16

HRMS spectrum of **BOD–CN** + ClO^{-} and **BOD–AL**……………………………………………17

1. Preparation of Reactive Oxygen Species (ROS)[1]

Preparation of hypochlorite (ClO^-)

The stock solution of sodium hypochlorite was purchased from Sigma-Aldrich Chemical Co. The concentration of ClO^- was determined by titration with $S_2O_3^{2-}$.

Preparation of hydrogen peroxide (H_2O_2)

The stock solution of H_2O_2 was purchased from Sigma-Aldrich Chemical Co. Proper concentration was prepared by dissolving in ultra-pure water.

Preparation of tert-butyl hydroperoxide (TBHP)

The stock solution of TBHP was purchased from Sigma-Aldrich Chemical Co. Proper concentration was prepared by dissolving in ultra-pure water.

Preparation of peroxyl radical (ROO•)

ROO• was generated from 2,2'-azobis(2-amidinopropane)dihydrochloride (AAPH). AAPH was dissolved in ultra-pure water and stirred at 25 °C for 30 min.

Preparation of hydroxyl radical (•OH)

Hydroxyl radical was generated by the Fenton reaction. To prepare •OH solution, ferrous chloride was added into the solution containing 10 equivalents of H_2O_2.

Preparation of superoxide radical ($O_2 \bullet^-$)

$O_2 \bullet^-$ radical was generated by dissolving potassium dioxide (KO_2) in DMSO.

Reference

[1] Z. N. Sun, F. Q. Liu, Y. Chen, P. K. H. Tam, D. Yang, A Highly Specific BODIPY-Based Fluorescent Probe for the Detection of Hypochlorous Acid, Org. Lett. 10 (2008) 2171– 2174. https://doi.org/10.1021/ol800507m.

2. Fluorescence Spectrum of BOD–CN with Different Solvent Systems

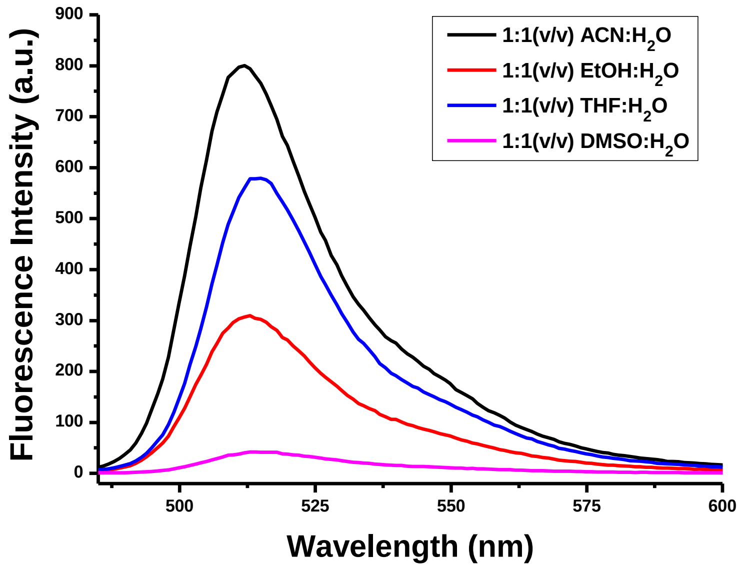

Figure S1. Fluorescence spectrum of **BOD–CN** (2.5 µM) in different solvent systems with ClO^{-} (20 equiv.).

3. Effect of Phosphate Buffer Solution (PBS) Content in Sensing Mechanism

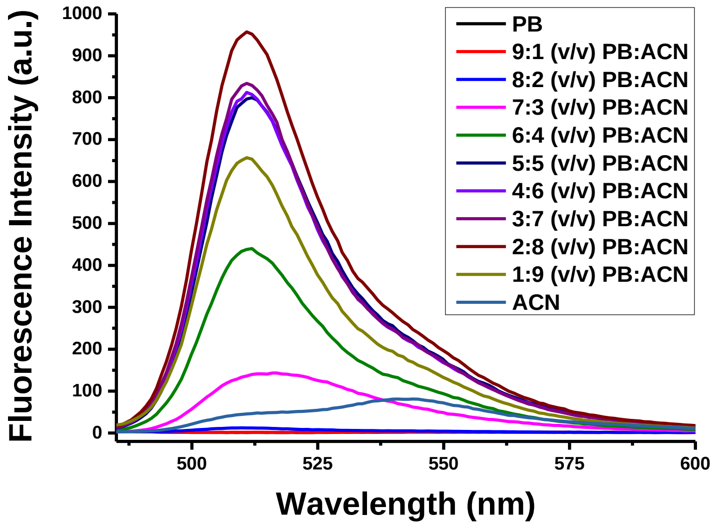

Figure S2. Effect of PBS content in the presence of **BOD–CN** (2.5 µM) and ClO^{-} (20 equiv.).

4. Effect of pH

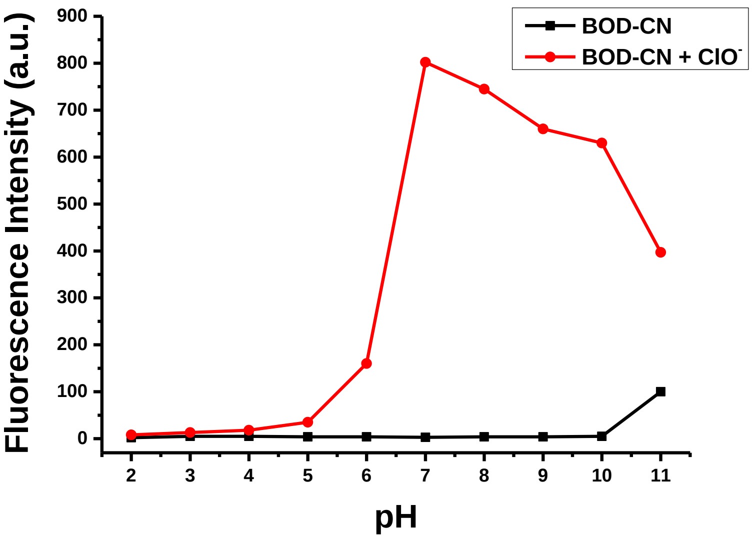

Figure S3. Effect of pH on the fluorescence intensity of **BOD–CN** (2.5 μM) in 1:1 CH_3CN/PBS in the absence (black line) and presence (red line) of $ClO^−$ (20 equiv.).

5. Time-Dependant Fluorescence Change of BOD–CN with ClO^-

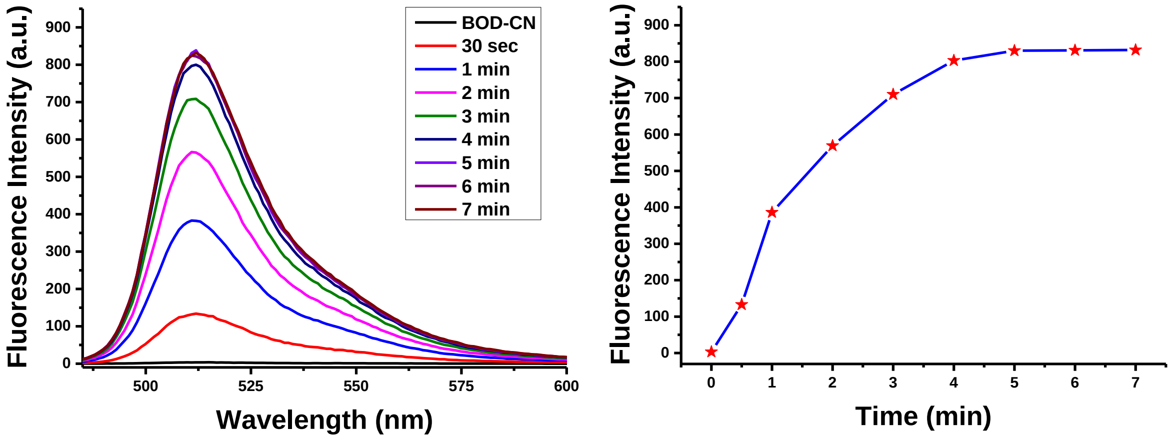

Figure S4. Time-dependent fluorescence change of **BOD–CN** (2.5 μM) in the presence of 20 equiv. $ClO^−$, measured in 1:1 CH_3CN/PBS at pH = 7.4.

6. LOD Determination of ClO^-

The detection limit was calculated based on fluorescence titration. The fluorescence emission intensity of **BOD–CN** (2.5 µM) in the absence of ClO^- was measured 10 times, and then, the standard deviation of blank measurements was determined. A good linear relationship between the fluorescence intensity and ClO^- concentration was obtained between 0.1 – 0.5 µM. The detection limit was then calculated using the equation: detection limit = 3.29σbi/m, where σbi is the standard deviation of sample measurements; m is the slope between intensity and sample concentration. The detection limit was measured to be 83.3 nM by regression data analysis.

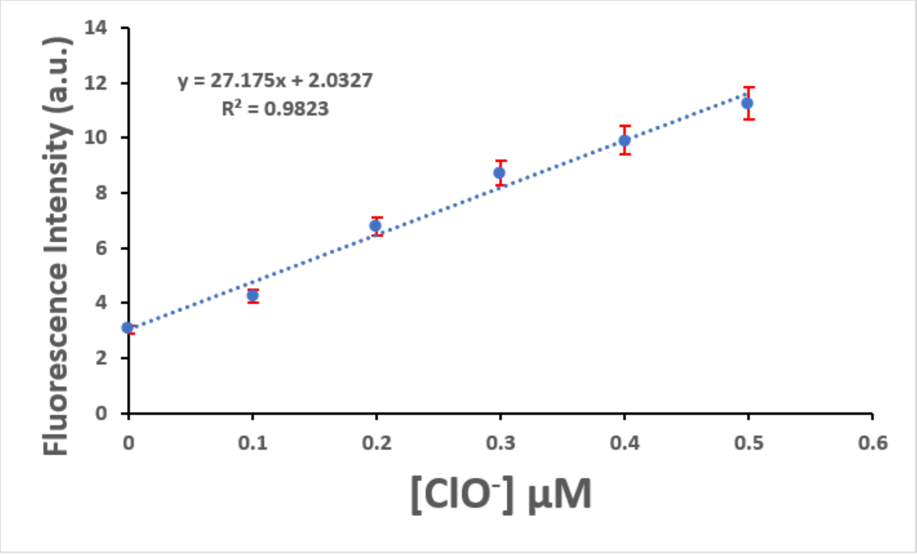

Figure S5. Fluorescence changes of **BOD–CN** (2.5 µM) upon addition of ClO^- (0.1 µM to 0.5 µM, 0.04 to 0.2 equiv.).

7. Calibration Curves of Real Water Samples and Disinfectant

a)

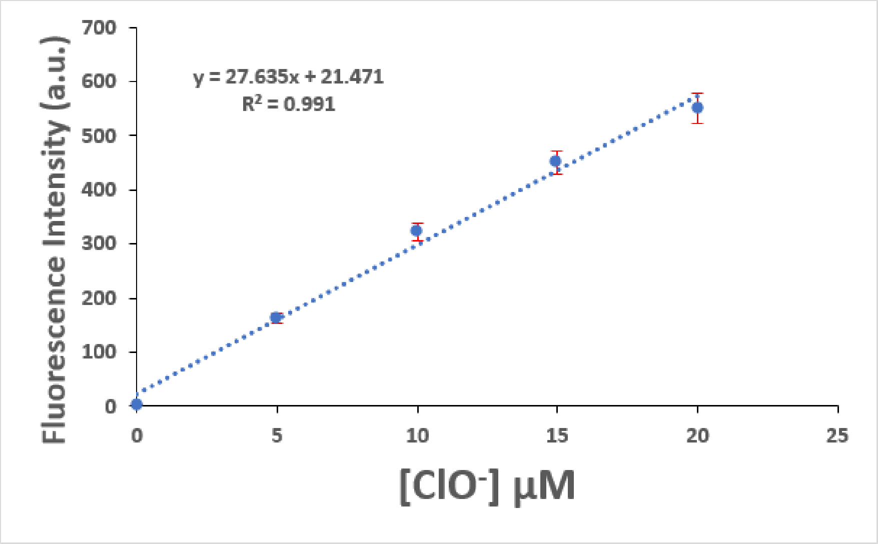

b)

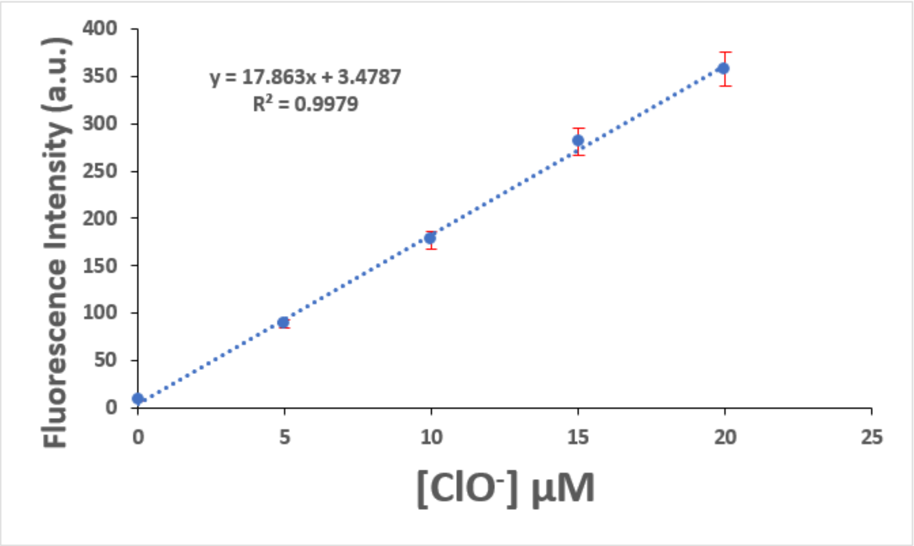

c)

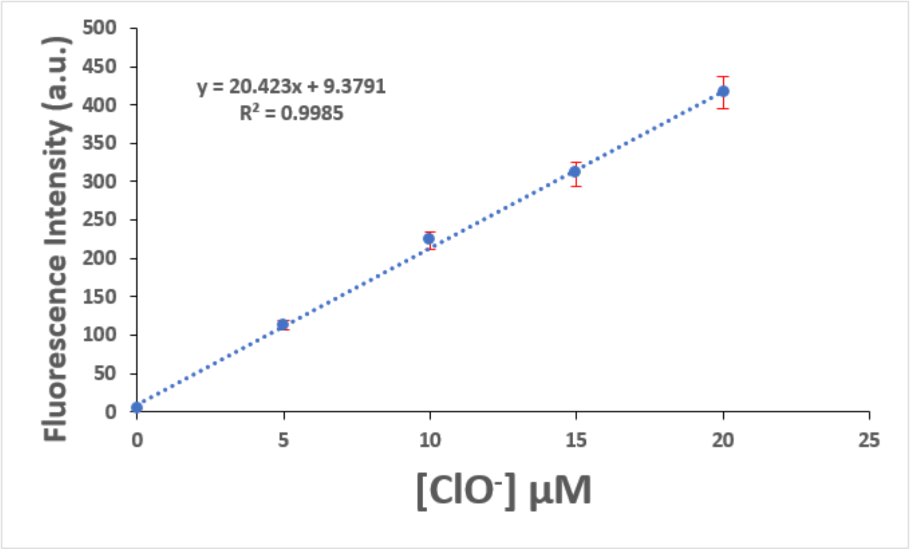

d)

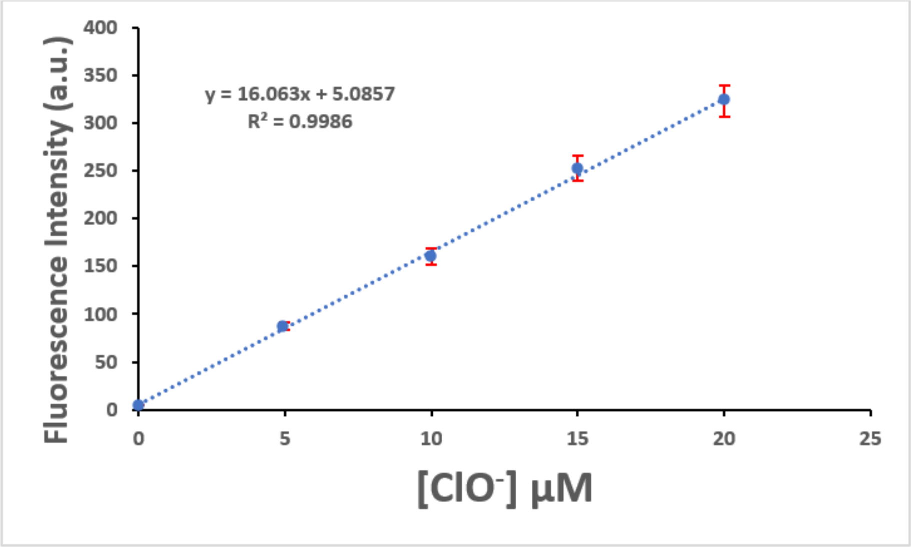

Figure S6. Calibration curves of **(a)** spring water, **(b)** wastewater, **(c)** pool water, **(d)** diluted disinfectant for ClO^-.

8. Calibration Curves of Fruit and Vegetable Samples

a)

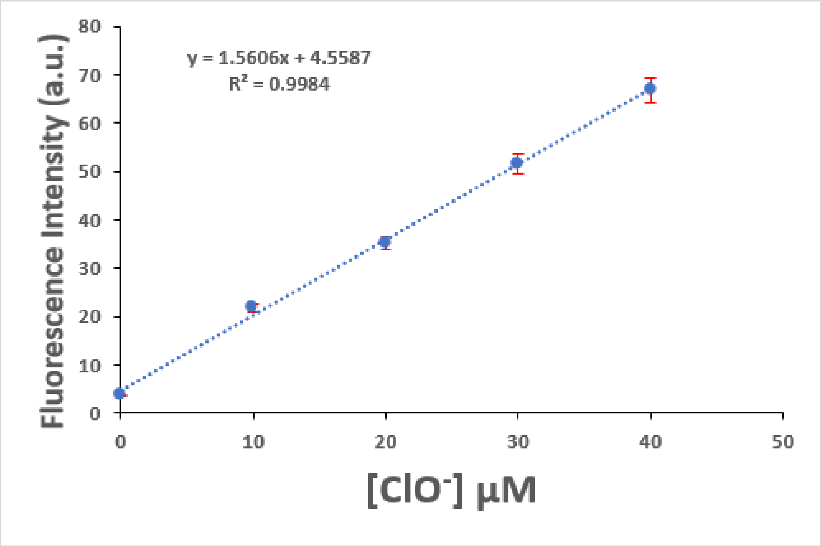

b)

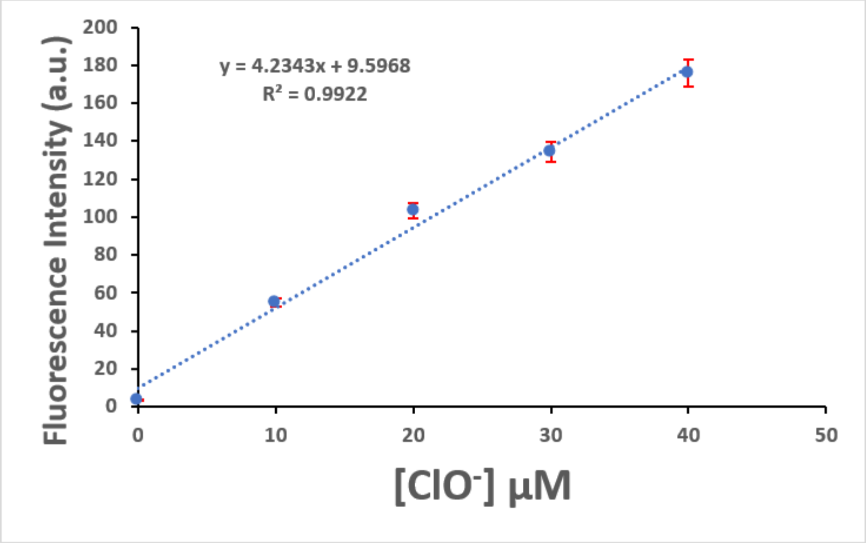

c)

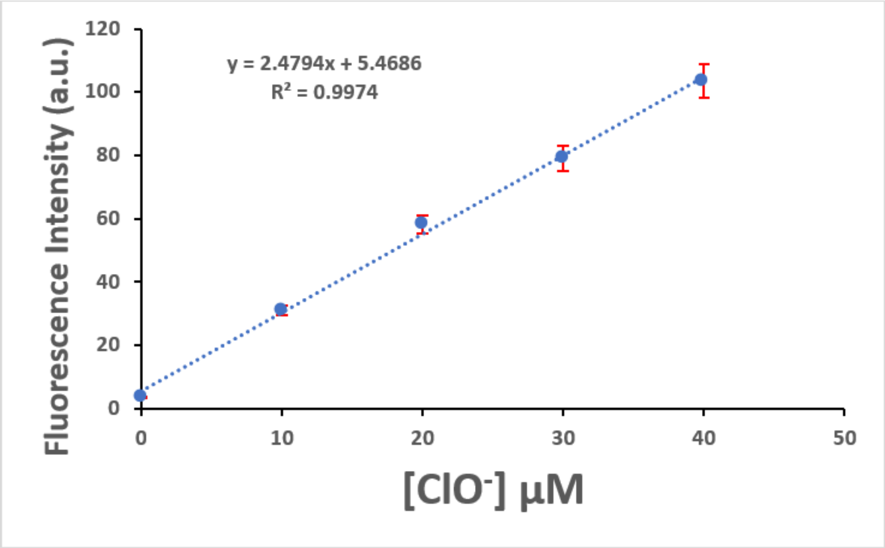

d)

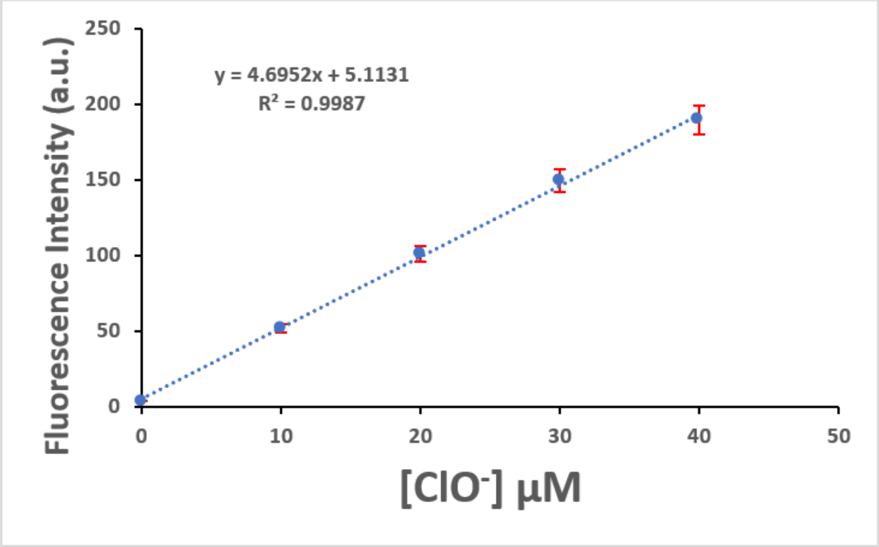

e)

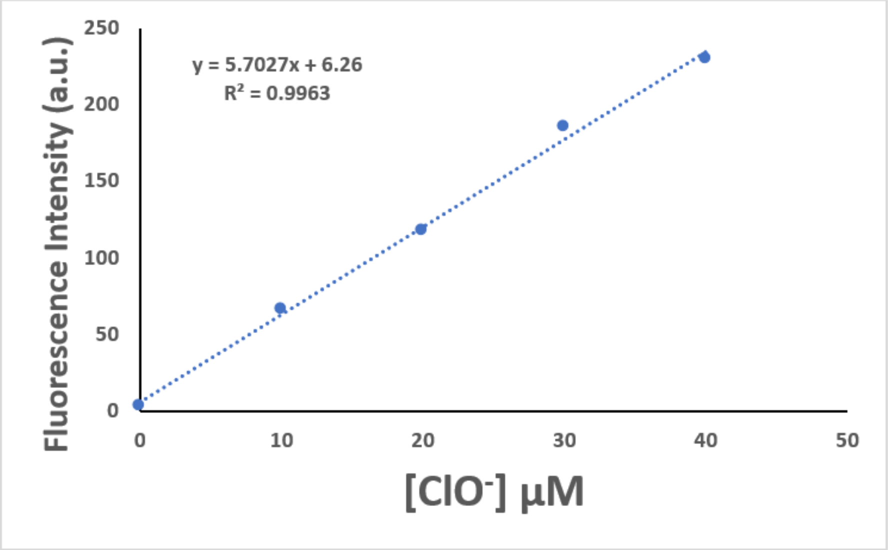

Figure S7. Calibration curves of **(a)** strawberry, **(b)** tomato, **(c)** cucumber, **(d)** lettuce, **(e)** spinach samples for ClO^{-}.

9. Calibration Curves of Dairy Products

a)

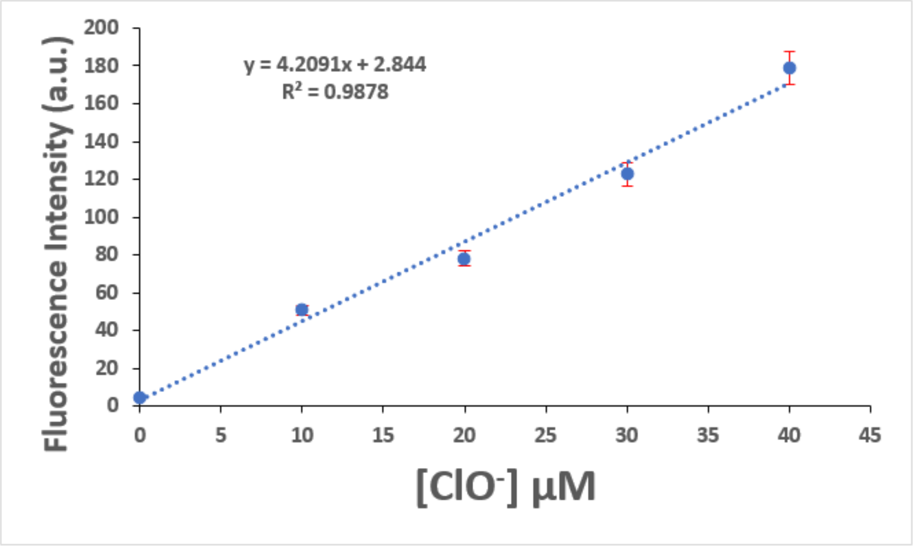

b)

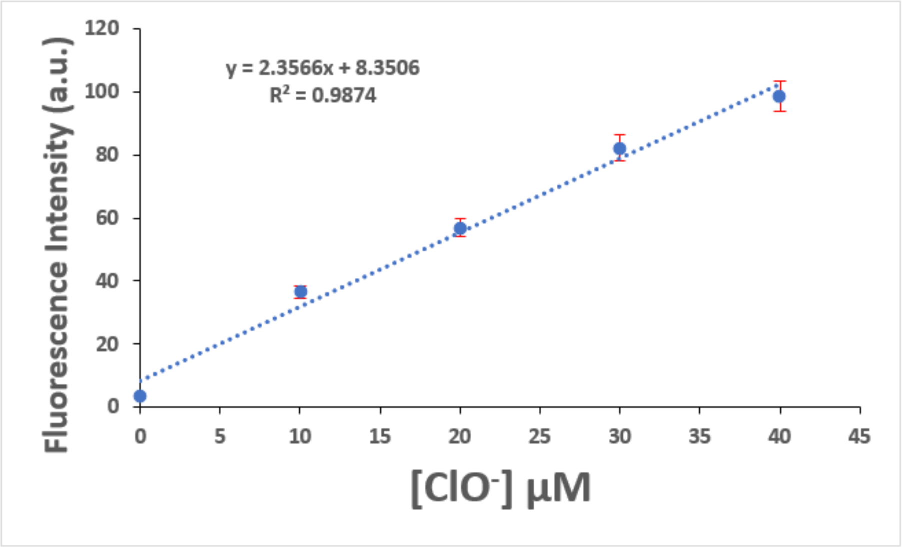

Figure S8. Calibration curves of **(a)** milk, **(b)** yogurt products for ClO^{-}.

10. Comparison Table with Other Reported ClO⁻ Fluorescent Probes

Table 1. Comparison Table with Other Reported ClO⁻ Fluorescent Probes

Compound	Mechanism	Solvent System	Response Time	LOD	Application	Ref. No[a]
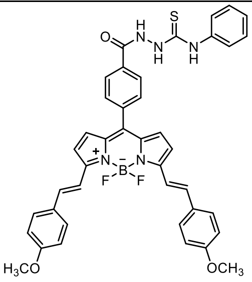	Ratiometric	1:1 THF/ PBS	10 min	1.15 μM	Cell imaging	[1]
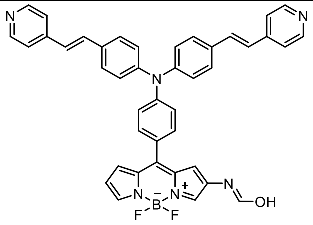	Turn-on	1:1 THF/ PBS	5 min	0.73 μM	Water sample	[2]
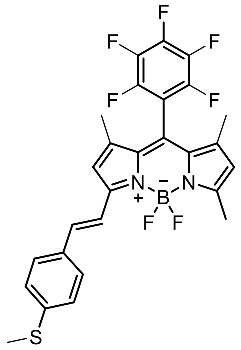	Ratiometric	1:1 DMF/ PBS	< 30 sec	59 nM	Cell, zebrafish and mouse imaging	[3]
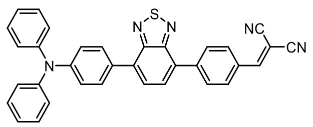	Turn-on	THF	~2 min	75 nM	Cell imaging	[4]
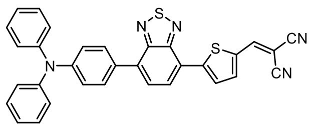	Turn-on	THF	~1 min	33 nM	Cell imaging	[4]
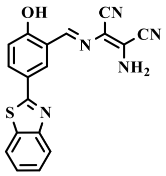	Turn-on	7:3 (v/v) DMSO/ H_2O	1 min	136 nM	Cell imaging, Water sample	[5]

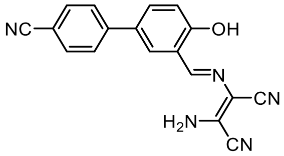	Ratiometric	HEPES	30 s	0.33 μM	Cell imaging, Water sample	[6]
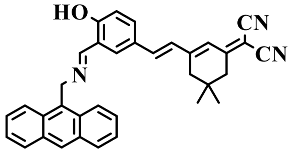	Ratiometric	8:2 (v/v) DMF/ PBS	5 min	0.29 μM	Cell imaging	[7]
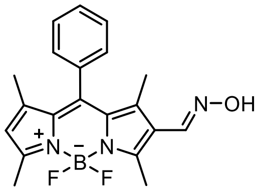	Turn-on	4:1 (v/v) PB/DMF	5 min	0.5 μM	Cell imaging	[8]
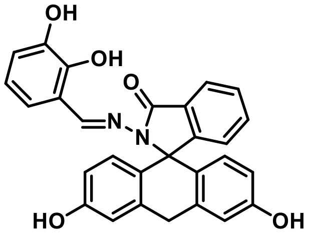	Ratiometric	7:3 (v/v) H_2O/THF	15 s	36.3 nM	Paper strips, water samples and potato sprouts	[9]
Curcumin/TPE@HyNPs	Ratiometric	PBS	140 s	0.35 μM	Milk samples	[10]
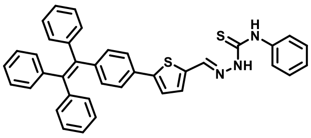	Turn-off	5:95 (v/v) THF/PBS	30 s	2 nM	Water samples & dairy products	[11]
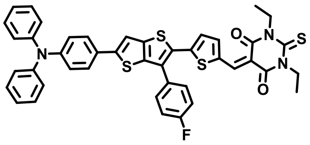	Turn-on	THF	4 min	0.27 μM	Water, fruit vegetable samples	[12]
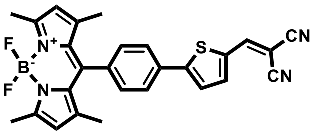	Turn-on	1:1 (v/v) ACN/PBS	< 30 s	83.3 nM	Water, fruit and vegetable samples & dairy products	This work

References

[1] B. Shen, Y. Qian, Z. Qi, C. Lu, Q. Sun, X. Xia, Y. Cui, Near-infrared BODIPY-based two-photon ClO^- probe based on thiosemicarbazide desulfurization reaction: naked-eye detection and mitochondrial imaging, J. Mater. Chem. B. 5 (2017) 5854–5861. https://doi.org/10.1039/C7TB01344B.

[2] X. Xu, Y. Qian, A novel pyridyl triphenylamine–BODIPY aldoxime: Naked-eye visible and fluorometric chemodosimeter for hypochlorite, Spectrochim. Acta Part A Mol. Biomol. Spectrosc. 183 (2017) 356–361. https://doi.org/https://doi.org/10.1016/j.saa.2017.04.043.

[3] C. Duan, M. Won, P. Verwilst, J. Xu, H. S. Kim, L. Zeng and J. S. Kim, In Vivo Imaging of Endogenously Produced HClO in Zebrafish and Mice Using a Bright, Photostable Ratiometric Fluorescent Probe, Anal. Chem 91 (2019) 4172. https://doi.org/10.1021/acs.analchem.9b00224

[4] L. Wang, R. Zhang, Y.-C. Bu, Z. Huang, L. Kong, J.-X. Yang, Two novel “turn on” fluorescent probes for monitoring hypochlorite in living HeLa cells, Dye. Pigment. 196 (2021) 109749. https://doi.org/https://doi.org/10.1016/j.dyepig.2021.109749.

[5] S. Malkondu, S. Erdemir, S. Karakurt, Red and blue emitting fluorescent probe for cyanide and hypochlorite ions: Biological sensing and environmental analysis, Dye. Pigment. 174 (2020) 108019. https://doi.org/https://doi.org/10.1016/j.dyepig.2019.108019.

[6] X. Tang, Z. Zhu, R. Liu, Y. Tang, A novel ratiometric and colorimetric fluorescent probe for hypochlorite based on cyanobiphenyl and its applications, Spectrochim. Acta Part A Mol. Biomol. Spectrosc. 219 (2019) 576–581. https://doi.org/https://doi.org/10.1016/j.saa.2019.04.042.

[7] S.-S. Liu, J.-L. Yan, W.-N. Wu, X.-L. Zhao, Y.-C. Fan, Y. Wang, Z.-H. Xu, Highly selective fluorescent probe for rapid turn-on detection and cell imaging of hypochlorite anion, J. Photochem. Photobiol. A Chem. 432 (2022) 114082. https://doi.org/https://doi.org/10.1016/j.jphotochem.2022.114082.

[8] M. Emrullahoğlu, M. Üçüncü, E. Karakuş, A BODIPY aldoxime-based chemodosimeter for highly selective and rapid detection of hypochlorous acid, Chem. Commun. 49 (2013) 7836–7838. https://doi.org/10.1039/C3CC44463E.

[9] M.A. Assiri, M.T. Waseem, A. Hamad, M. Imran, U. Farooq, S.A. Shahzad, Ratiometric and colorimetric probes with large stokes shift for sensing of exogenous hypochlorite in potato sprouts and industrial effluents, Spectrochim. Acta Part A Mol. Biomol. Spectrosc. 290 (2023) 122298. https://doi.org/https://doi.org/10.1016/j.saa.2022.122298.

[10] Y. Shen, C. Nie, C. Zhu, Z. Zheng, Y. Wu, Aggregation-Induced Emission Fluorophore-Incorporated Curcumin-Based Ratiometric Nanoprobe for Hypochlorite Detection in Food Matrices, J. Agric. Food Chem. 70 (2022) 9577–9583. https://doi.org/10.1021/acs.jafc.2c03826.

[11] E. Erdemir, G. Suna, S. Gunduz, M. Şahin, S. Eğlence-Bakır, E. Karakuş, Tetraphenylethylene–thiosemicarbazone based ultrafast, highly sensitive detection of hypochlorite in aqueous environments and dairy products, Anal. Chim. Acta. 1218 (2022) 340029. https://doi.org/https://doi.org/10.1016/j.aca.2022.340029.

[12] G. Suna, S. Gunduz, S. Topal, T. Ozturk, E. Karakuş, A unique triple–channel fluorescent probe for discriminative detection of cyanide, hydrazine, and hypochlorite, Talanta. 257 (2023) 124365. https://doi.org/https://doi.org/10.1016/j.talanta.2023.124365.

^{1}H and ^{13}C NMR of BOD–AL in CDCl$_3$

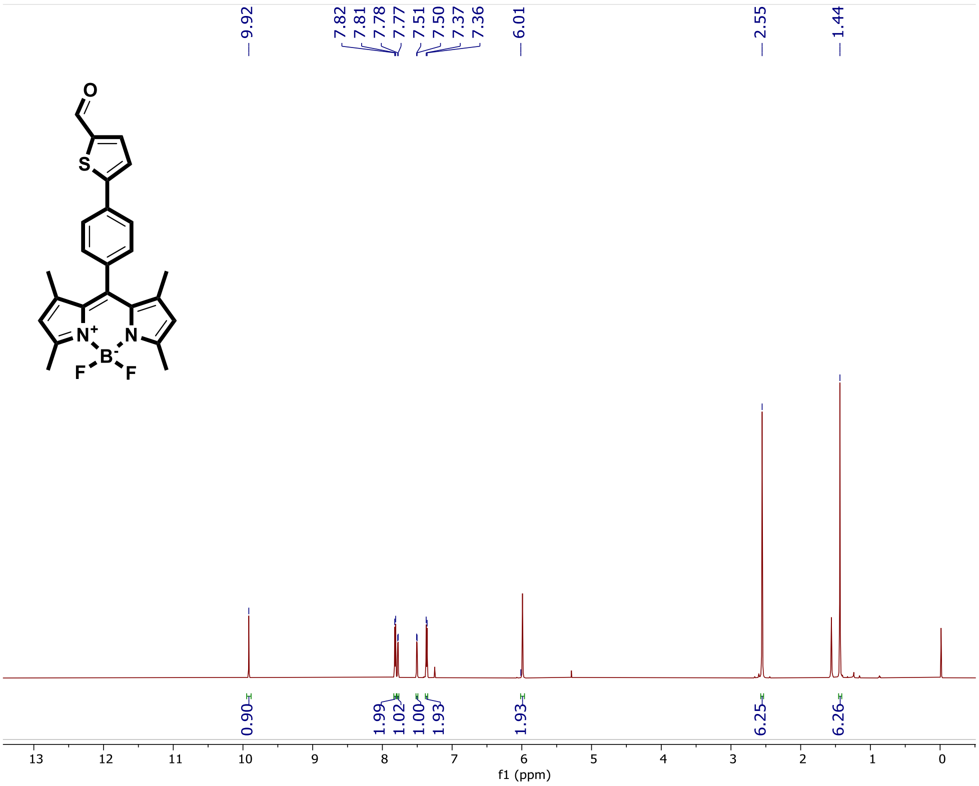

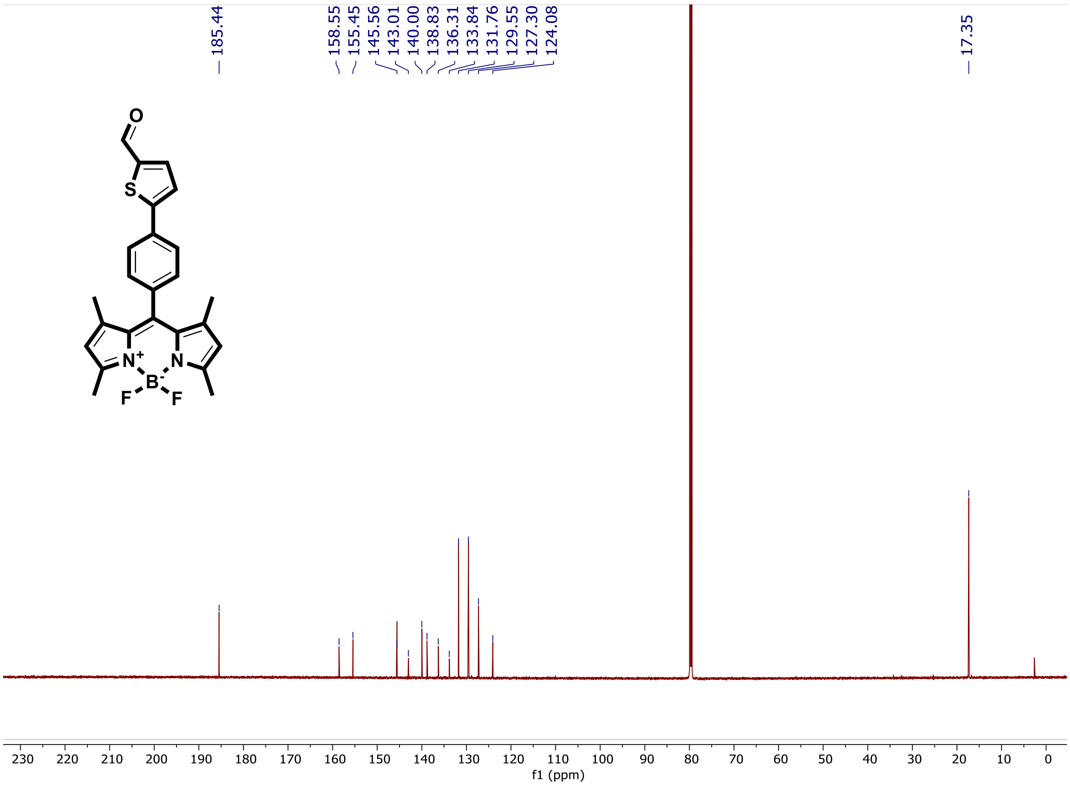

Figure S9. ^{1}H and ^{13}C NMR spectra of **BOD–AL** in CDCl$_3$

^1H and ^{13}C NMR of BOD–CN in $CDCl_3$

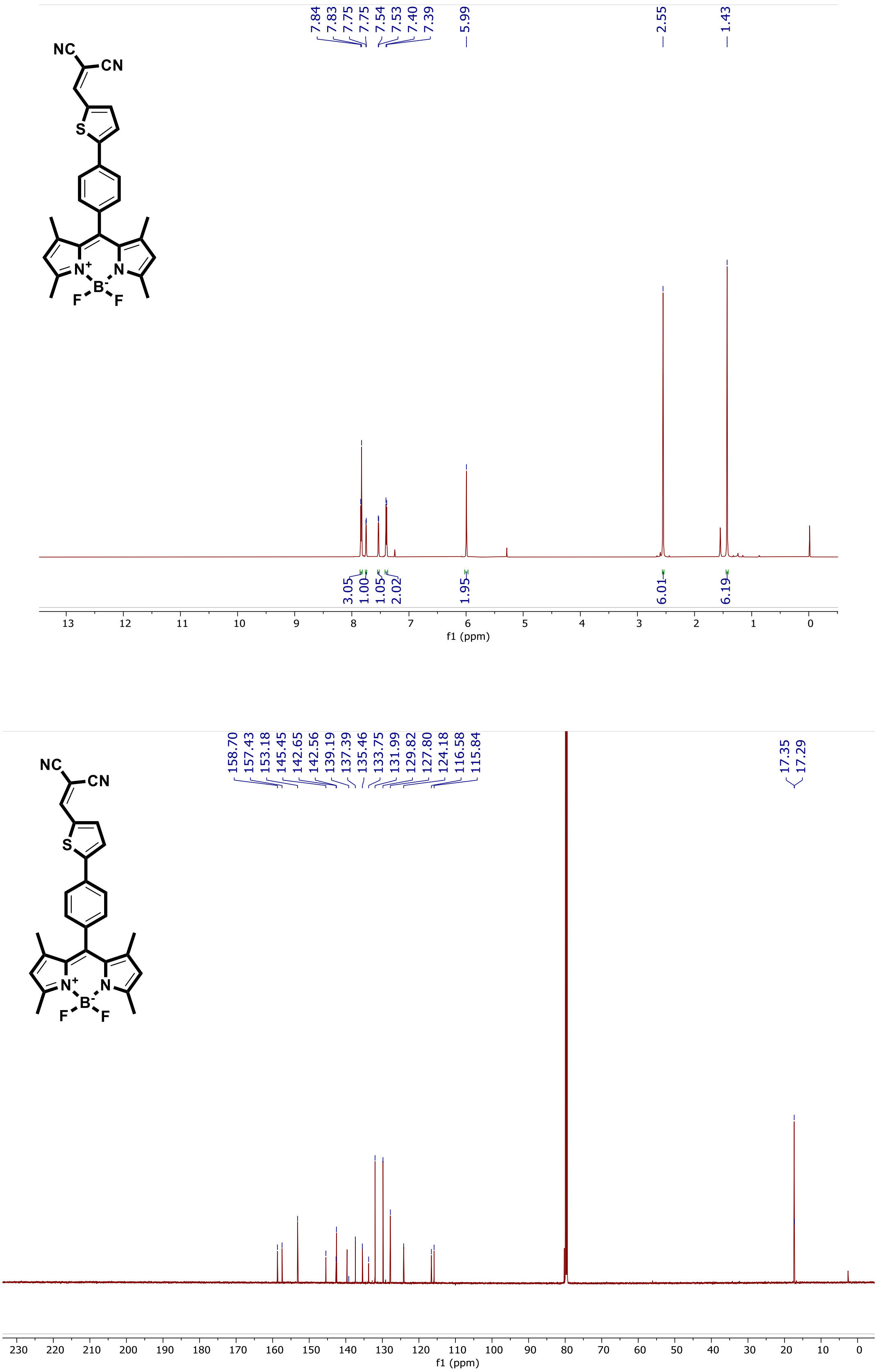

Figure S10. ^1H and ^{13}C NMR spectra of **BOD–CN** in $CDCl_3$

^{1}H and ^{13}C NMR of BOD–AL in $CDCl_3$ after oxidation with ClO^-

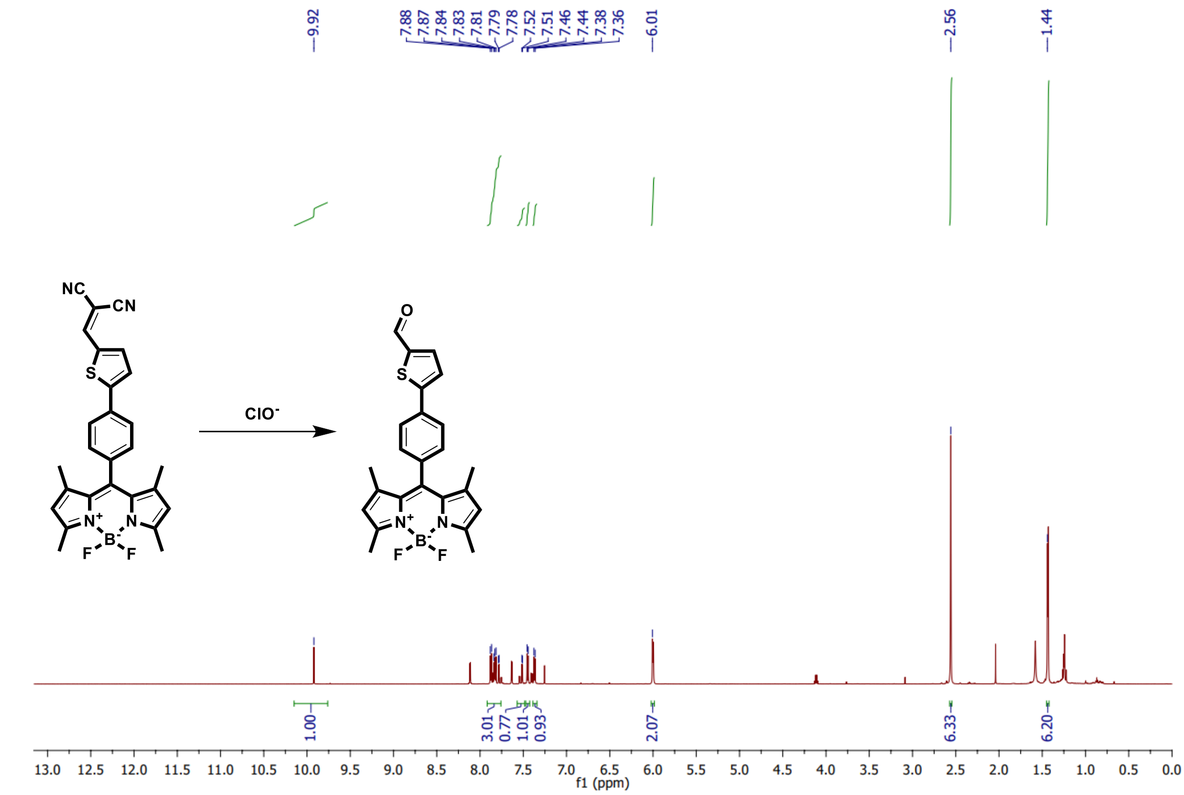

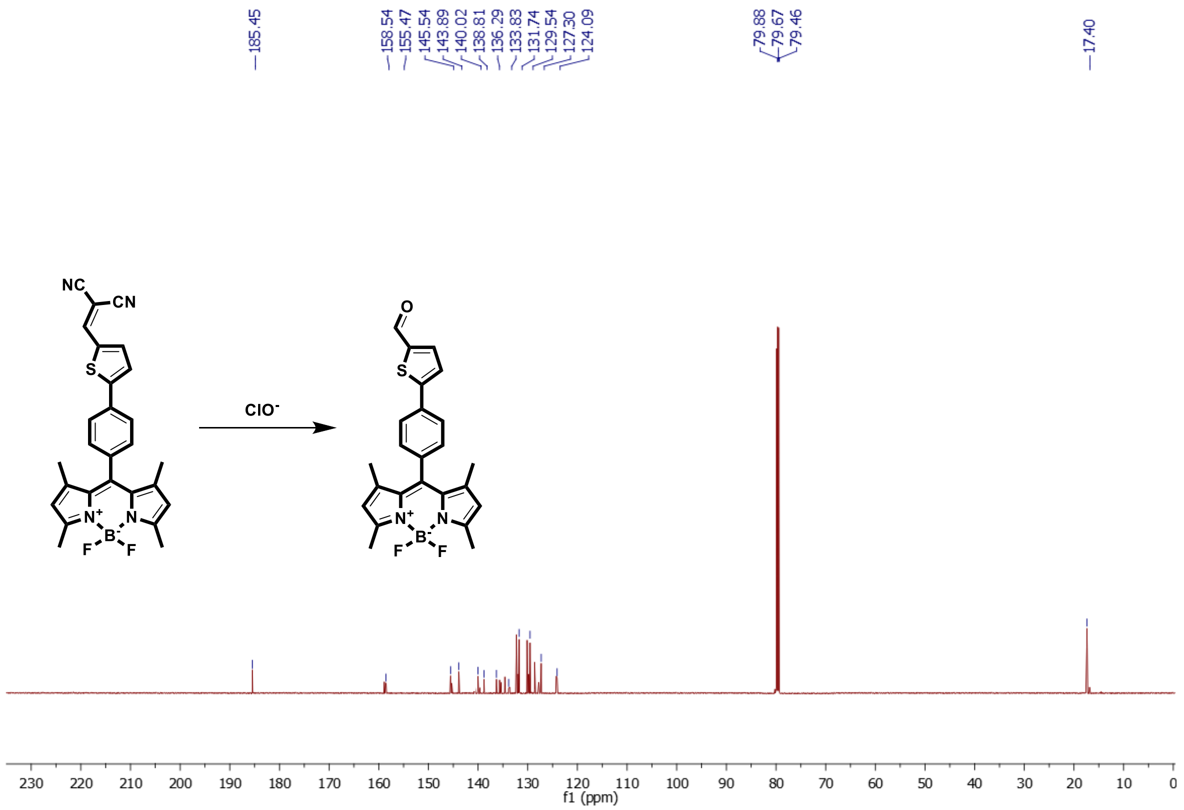

Figure S11. ^{1}H and ^{13}C NMR spectra of **BOD–AL** in $CDCl_3$ after oxidation

HRMS spectrum of BOD–AL

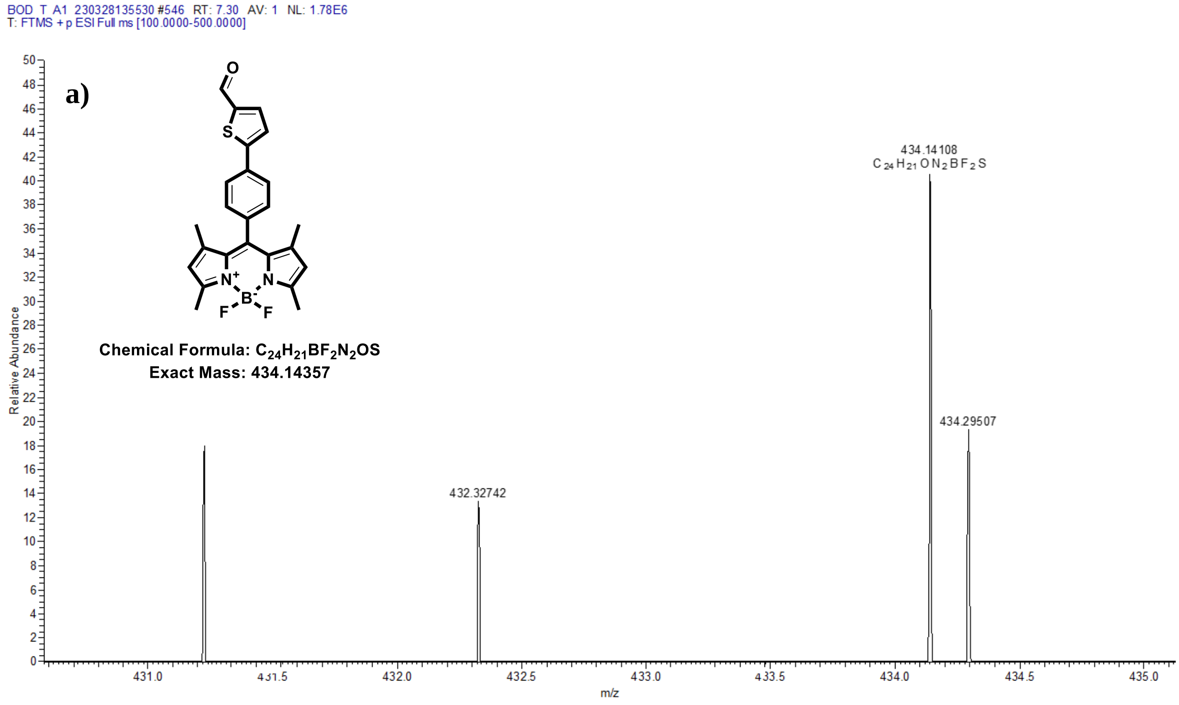

HRMS spectrum of BOD–CN

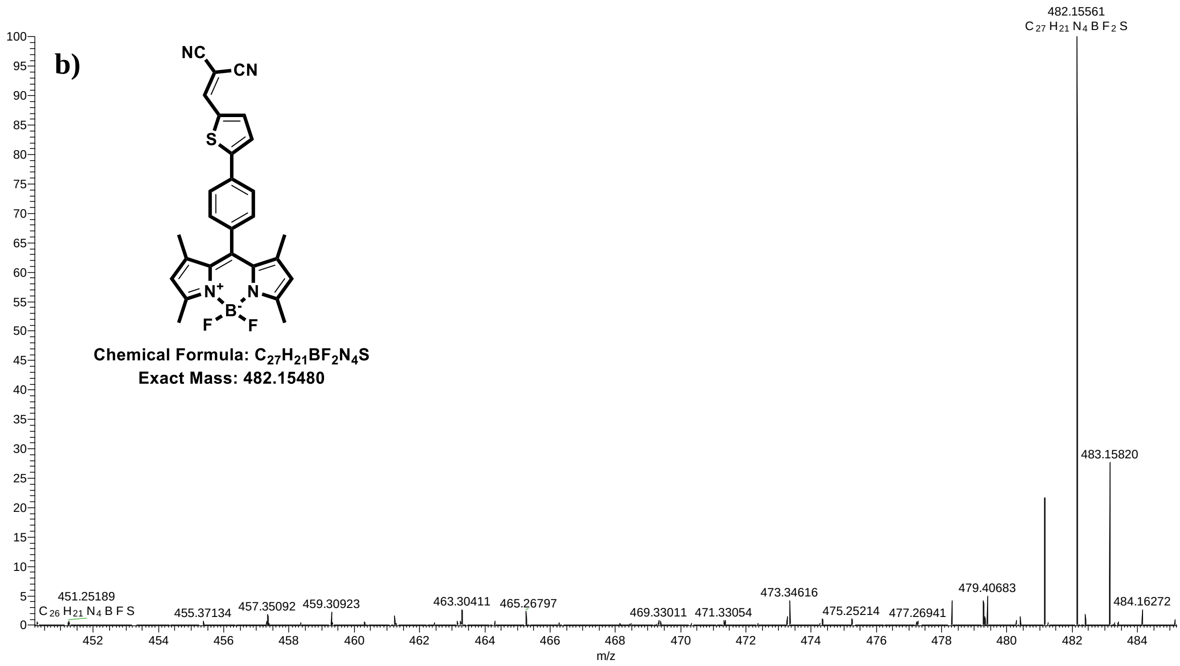

HRMS spectrum of BOD–CN + ClO⁻

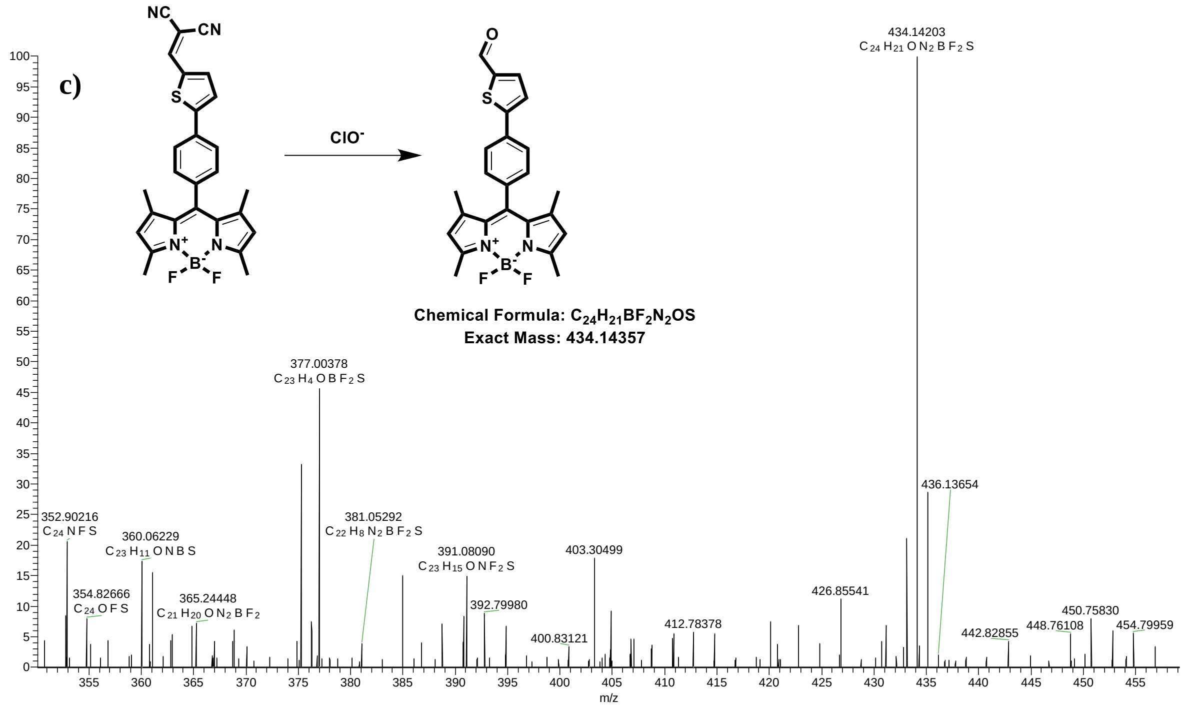

HRMS spectrum of BOD–AL after oxidation reaction

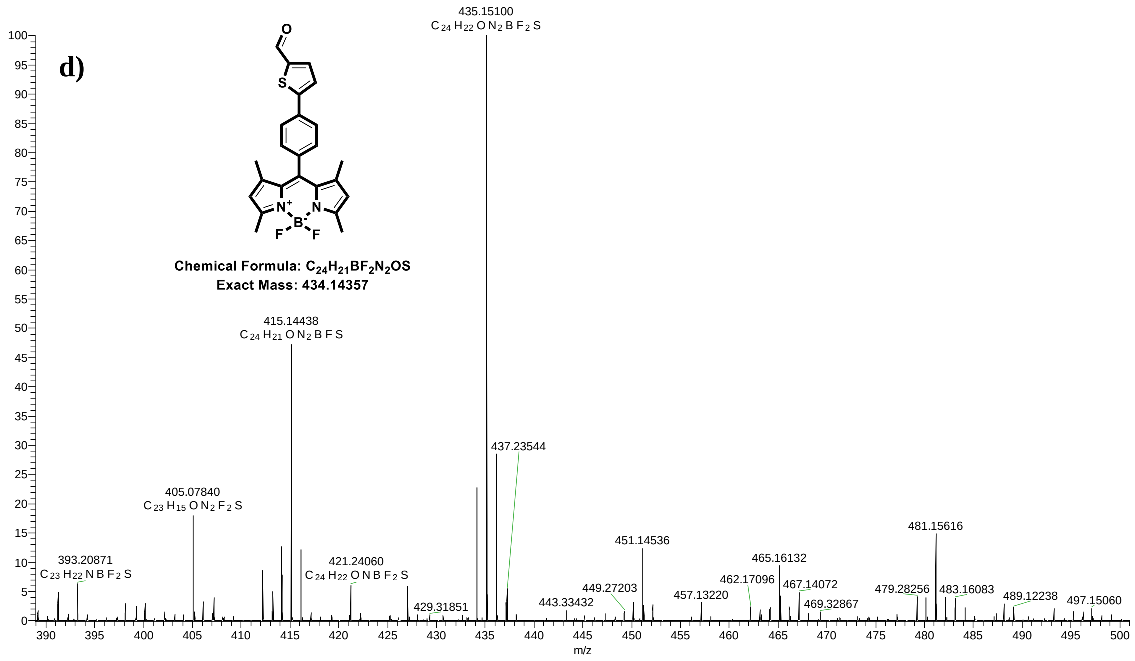

Figure S12. HRMS spectrum of **(a) BOD–AL, (b) BOD–CN**, **(c) BOD–CN +** ClO⁻ and **(d) BOD–AL** after oxidation reaction
